# Characterization and Complete Genomic Analysis of a Novel Bacteriophage BUCT775 for *Acinetobacter baumannii* and Its Elimination Efficiency in the Environment

**DOI:** 10.3390/ijms26157279

**Published:** 2025-07-28

**Authors:** Yuxuan Liu, Yunfei Huang, Dongxiang Zhu, Lefei Zhang, Jianwei Zhang, Yigang Tong, Mengzhe Li

**Affiliations:** 1 College of Chemical Engineering, Beijing University of Chemical Technology, Beijing 100029, China; liuyuxuanmail@163.com; 2 State Key Laboratory of Green Biomanufacturing, College of Life Science and Technology, Beijing University of Chemical Technology, Beijing 100029, China; huangyunfei2000@163.com (Y.H.); 2023201242@buct.edu.cn (L.Z.); 3 College of Humanities and Law, Beijing University of Chemical Technology, Beijing 100029, China; zhudx@buct.edu.cn (D.Z.); zhangjianweimail@163.com (J.Z.)

**Keywords:** *Acinetobacter baumannii*, characterization, genomic analysis, phage therapy, phage disinfection

## Abstract

*Acinetobacter baumannii* (*A. baumannii*) is an opportunistic pathogen responsible for a range of severe infections and nosocomial outbreaks. Phage-based therapy and biocontrol represent effective strategies to combat the prevalence of *A. baumannii*. This study reports a novel phage, BUCT775, capable of specifically lysing *A. baumannii*, and investigates its physiological properties, genomic characteristics, in vivo therapeutic efficacy, and environmental disinfection performance. Phage BUCT775 is a podovirus that forms clear, well-defined plaques with an average diameter of 2.5 ± 0.52 mm. It exhibits a broad range of temperature stability (4–55 °C) and pH stability (pH 3–12). The optimal multiplicity of infection (MOI) for phage BUCT775 is 0.01. At an MOI of 0.01, it demonstrates a latent period of approximately 10 min and exhibits a high burst size. Genomic sequencing and bioinformatics analysis revealed that phage BUCT775 belongs to the order *Caudoviricetes* and the family *Autographiviridae*. Its genome has a G + C content of 39.3% and is not known to contain virulence genes or antibiotic resistance genes. Phage BUCT775 exhibited significant therapeutic effects on *A. baumannii*-infected *G. mellonella* larvae, increasing the 120 h survival rate of the larvae by 20%. Additionally, phage BUCT775 efficiently eliminated *A. baumannii* in the environment, with an average clearance rate exceeding 98% within 3 h. These studies suggest that phage BUCT775 holds significant potential for application in phage therapy and environmental disinfection.

## 1. Introduction

*Acinetobacter baumannii* (*A. baumannii*) is a Gram-negative and ubiquitous pathogen that plays a significant role in various human infections, including pneumonia, bacteremia, soft tissue, and urinary tract infections [1,2,3,4]. It is an opportunistic pathogen and a major cause of nosocomial infections, especially in intensive care units (ICUs) [5,6,7,8]. The adhesive properties, colonization ability, and desiccation resistance of *A. baumannii* play a critical role in its persistence and transmission within hospital environments [6,9,10].

The overuse and misuse of antibiotics have enabled *A. baumannii* to develop multiple antibiotic resistance mechanisms, leading to an increase in the prevalence of resistant strains and posing a significant challenge in clinical settings [2,11,12,13,14]. The global spread of multidrug-resistant *A. baumannii* is alarming, with the overall mortality rate for *A. baumannii* pneumonia estimated up to 43% [15,16]. In response to this growing threat, the World Health Organization (WHO) designated carbapenem-resistant *A. baumannii* (CRAB) as a critical priority in its Bacterial Priority Pathogens List (BPPL) [17]. In China, the proportion of CRAB has increased rapidly in recent years, causing a substantial clinical and economic burden on the healthcare system [16,18].

Bacteriophages (phages) are viruses that specifically infect bacteria and are considered to be the most abundant and ancient organisms on Earth [19]. Phages have relatively small genomes, exhibit strict host specificity, and rely on the hosts’ machinery for replication [20]. Since their discovery over a century ago, phages have been explored as potential therapeutic agents for bacterial infections [21]. As the prevalence of drug-resistant bacterial strains continues to rise, phage therapy has emerged as a potent alternative to address the growing challenge of bacterial resistance, particularly for infections caused by multidrug-resistant pathogens [20,21]. Phage therapy can effectively control the CRAB infection rate in hospitals, and is more effective in removing bacterial biofilms and bacteria on hard surfaces when used in combination with chemical disinfectants [22,23].

To effectively combat the growing resistance crisis and provide targeted treatments, there is an urgent need to build a large, diverse phage library that can be tailored to the varying pathogens present in different patients. This library would enable the rapid identification and application of appropriate phages to combat specific bacterial infections. However, this requires the isolation of a wide range of phages from various sources to ensure comprehensive coverage of potential pathogens. In this study, we reported the isolation and characterization of phage BUCT775, a novel species infecting *A*. *baumannii*. The discovery of BUCT775 enriched the diversity of phages targeting clinically drug-resistant strains and expanded the current phage library for combating multidrug-resistant *A. baumannii*. We systematically evaluated its biological properties and genomic features, highlighting its potential for therapeutic application and hospital environmental disinfection.

## 2. Results and Discussion

### 2.1. Morphology

**Figure 1:**
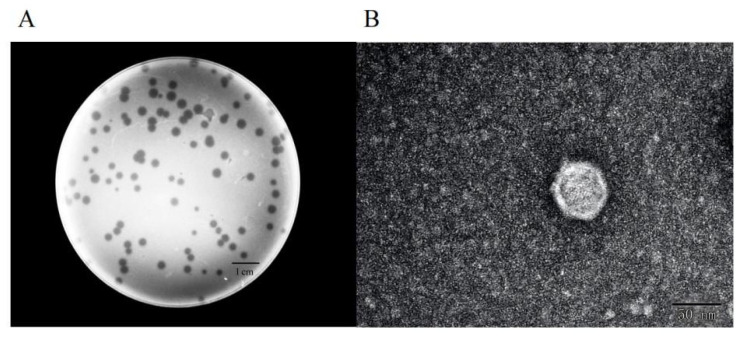
Morphological characteristics of phage BUCT775: (**A**) Phage plaques formed on the lawn of *A. baumannii* strain 3808. (**B**) Transmission electron micrograph of phage BUCT775.

Using *A. baumannii* strain 3808 as the host, we isolated a phage from wastewater and named it BUCT775. Phage BUCT775 formed clear and transparent plaques on the bacterial lawn of its host, with a diameter of 2.5 ± 0.52 mm and regular edges (Figure 1A). The transmission electron microscopy (TEM) images revealed that phage BUCT775 has an average head diameter of 55.84 ± 1.74 nm and an average tail length of 12.31 ± 1.51 nm. Based on these characteristics, it could be classified as a member of podovirus, exhibiting a small and non-contractile tail (Figure 1B).

### 2.2. Host Range

Determining the host range and sensitivity to different bacterial hosts is essential for evaluating their potential applications. In this study, we assessed the host range of phage BUCT775 by its infectivity against 15 different *A. baumannii* strains. The results showed that phage BUCT775 specifically infected only four ST2 *A. baumannii* strains with strong infectivity, indicating its high host specificity (Table 1). The efficiency of plating (EOP) was calculated as the ratio of the phage titer (PFU/mL) on the test host to that on the reference host. EOP analysis revealed that the efficiency of plating (EOP) values for strains T2634, T3666, and T3789 were 0.1, 1, and 1, respectively. The lytic efficiency of phage BUCT775 against the test strains is lower than that of pIsf-AB02 phage, ØABP-01, ØABP-02, and TCUP2199, suggesting that its host range may be narrower and that it exhibits higher host specificity [24,25,26].

### 2.3. Optimal Multiplicity of Infection and One-Step Growth Curve

**Figure 2:**
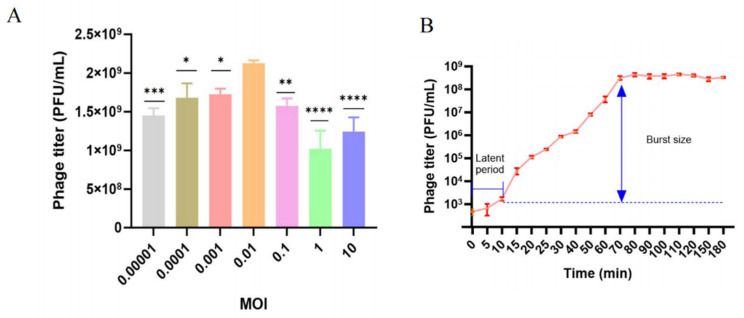
Multiplicity of infection and one-step growth curve of BUCT775: (**A**) Multiplicity of infection. (**B**) One-step growth curve of phage BUCT775. Data are presented as mean ± standard deviation. * *p* < 0.05, ** *p* < 0.01, *** *p* < 0.001, and **** *p* < 0.0001.

Phage BUCT775 was mixed with *A. baumannii* strain 3808 at different multiplicities of infection (MOIs) and incubated at 37 °C for 12 h. The phage titers were then determined using the soft agar overlay method. The results indicated that at an MOI of 0.01, phage titer reached about 2.12 × 10^9^ PFU/mL, which was significantly higher than that observed at other MOIs (Figure 2A). Therefore, an MOI of 0.01 was determined to be the optimal condition for phage BUCT775. The one-step growth curve of phage BUCT775 revealed a latent period of approximately 10 min, followed by a burst phase, which lasted for 60 min. At 70 min, the phage titer reached the plateau period, with its titer gradually stabilizing (Figure 2B). The average burst size was 335.7 ± 25.2 PFU/cell. Phage BUCT775, compared to pIsf-AB02 phage, ØABP-01, and ØABP-02, exhibits a shorter latent period and a higher burst size, indicating that it can proliferate and lyse host cells more rapidly [24,25].

### 2.4. Thermal and pH Stability of Phage BUCT775

**Figure 3:**
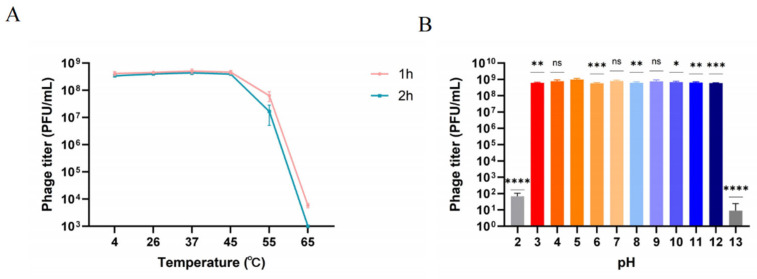
Thermal and pH stability of phage BUCT775: (**A**) Thermal stability of phage BUCT775. The stability of phage BUCT775 gradually decreases with increasing temperature. Red and green indicate the 1-hour and 2-hour stability of phage BUCT775 at different temperatures, respectively. (**B**) The pH stability of phage BUCT775. The titer of phage BUCT775 significantly decreases at pH 2 and pH 13. Data are presented as mean ± standard deviation and analyzed by one-way ANOVA followed by Dunnett’s multiple comparison test. * *p* < 0.05; ** *p* < 0.01; *** *p* < 0.001; **** *p* < 0.0001; ns, not significant.

The stability of phage BUCT775 was evaluated across a range of temperatures (4 °C, 26 °C, 37 °C, 45 °C, 55 °C, and 65 °C) over culture periods of 1 and 2 h. The phage titer was consistently lower after 2 h of incubation compared to 1 h at all temperatures. The highest titer was observed at 37 °C for both incubation times, while at 55 °C, the titer decreased nearly tenfold. The phage titer dropped significantly after 1 h of culture at 65 °C, and no detectable phage activity remained after 2 h (Figure 3A). These results indicated that phage BUCT775 was relatively stable at room temperature but was sensitive to high temperatures, with its stability sharply declining at elevated temperatures above 45 °C. The pH sensitivity of phage BUCT775 was also assessed by incubating the phage at pH values ranging from 3 to 12 for 1 h. The phages remained active within this pH range, with no significant changes observed between pH 4 and 12. However, when the pH was lower than 3 or higher than 12, the titer decreased markedly after 1 h of incubation (Figure 3B). These findings demonstrate that phage BUCT775 had strong pH tolerance to both extremely acidic and alkaline environments. The stability of phages is crucial for their applications across various fields [27]. Phage BUCT775 demonstrates excellent thermal stability, making it more suitable for storage and application. Additionally, it exhibits stability over a broader pH range, highlighting its potential as a biocontrol agent. In previous studies, phage vB_AbaM_AB3P2 retained robust activity at temperatures between 65 °C and 70 °C, whereas phage BUCT775 exhibited relatively lower heat tolerance [28]. Phage vB_AbaM_AB3P2 remained stable at pH 2 but lost activity at pH 11 [28]. In contrast, phage TCUP2199 lost activity at pH 3 and showed a significant decline in activity at pH 11 [26]. Compared with these two phages, phage BUCT775 demonstrated superior tolerance to alkaline environments.

### 2.5. Genomic and Phylogenetic Analysis of Phage BUCT775

**Figure 4:**
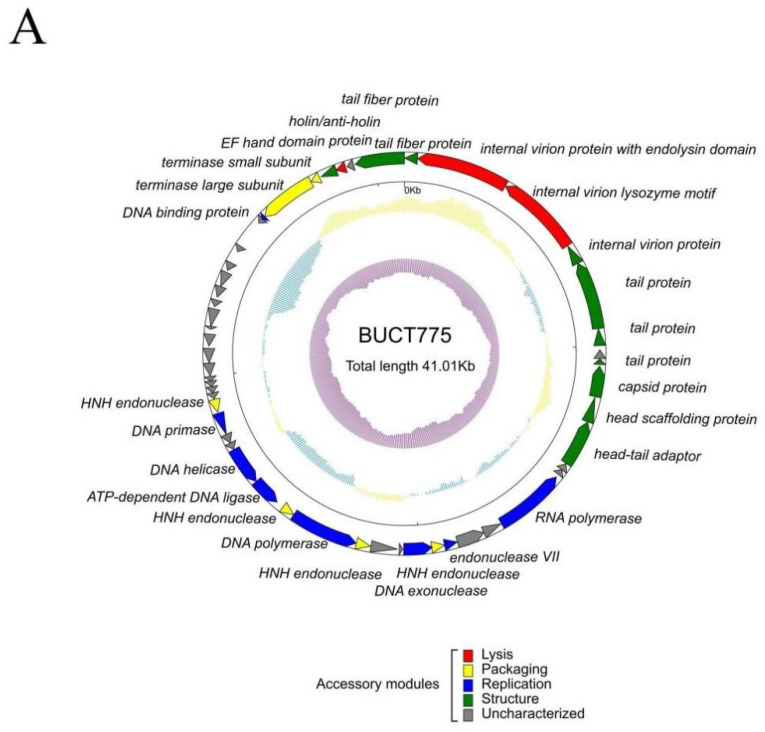

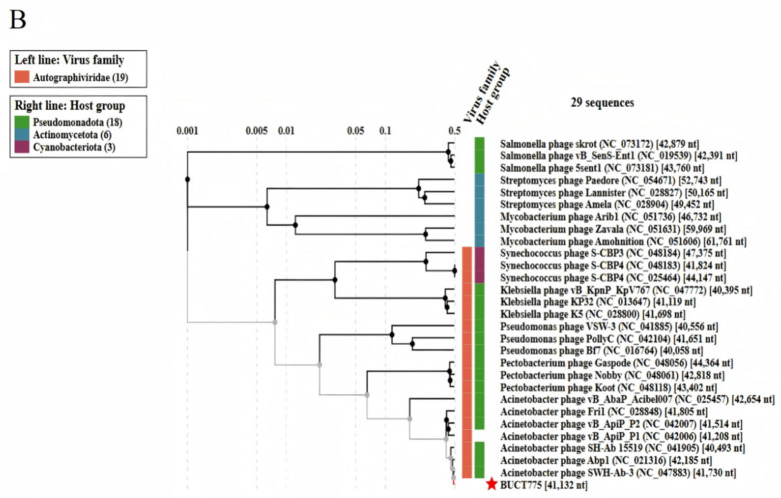
Genome map and phylogenetic analysis of phage BUCT775: (**A**) Genome map of phage BUCT775. The outer ring illustrates the open reading frames (ORFs), with various colors indicating different types of functional genes. The middle ring displays the (G + C) mol%, where yellow marks values above the genome-wide average, while blue shows values below it. The innermost purple ring represents the G − C/G + C skew, with inward-skewed sections indicating values less than 0. (**B**) Phylogenetic analysis of phage BUCT775. Construct a proteomic tree using the complete genome sequence of BUCT775 through the online tool ViPTree.

Phage BUCT775 contained double-stranded DNA with a total length of 41,005 bp and a GC content of 39% (Figure 4A). A BLAST (https://blast.ncbi.nlm.nih.gov/Blast.cgi, accessed on 9 July 2024) comparison of the phage BUCT775 genome against the NCBI database demonstrated that it had the highest similarity to *Acinetobacter* phage Abp1 (NC_021316.1), with a query cover of 95% and an identity of 98.60%. A total of 52 ORFs were identified in the genome of phage BUCT775, including 27 functional proteins and 25 hypothetical proteins (Table 2 and Table S1). Among the identified ORFs, ten were associated with structural components of the phage, including tail proteins (ORF 1, ORF 5, ORF 6, ORF 8, and ORF 52), internal virion protein (ORF 4), capsid protein (ORF 9), head scaffolding protein (ORF 10), and head–tail adaptor (ORF 11). Eight ORFs were involved in replication processes, including RNA polymerase (ORF 14), endonuclease VII (ORF 17), DNA exonuclease (ORF 19), DNA polymerase (ORF 23), ATP-dependent DNA ligase (ORF 25), DNA helicase (ORF 26), DNA primase (ORF 29), and DNA-binding protein (ORF 46). Six ORFs were related to packaging functions, including HNH endonucleases (ORF 18, ORF 22, ORF 24, and ORF 30), terminase large subunit (ORF 47), and terminase small subunit (ORF 48). Additionally, three ORFs were involved in lysis (ORF 2, ORF 3, and ORF 50). Phages that infect Gram-negative bacteria predominantly employ two distinct lysis mechanisms: the holin–endolysin and the pinholin-SAR endolysin pathways [29]. Genomic analysis revealed that the lytic mechanism of phage BUCT775 is primarily mediated by the holin protein encoded by ORF50. The endolysin encoded by ORF2 and the lysozyme encoded by ORF3 also contribute to host cell lysis. Furthermore, no known virulence or antibiotic resistance genes were annotated in the genome of phage BUCT775, ensuring its safety in applications.

To analyze the evolutionary relationship between phage BUCT775 and other phages, a proteomic tree was constructed using the online tool ViPTree. The analysis revealed that BUCT775 shared high homology with *Acinetobacter* phage SWH-Ab-3 (NC_047883), and it was classified within the order *Caudoviricetes*, the family *Autographiviridae,* and the subfamily *Beijerinckvirinae* (Figure 4B).

### 2.6. Antibacterial Effects of Phage BUCT775 In Vivo

**Figure 5:**
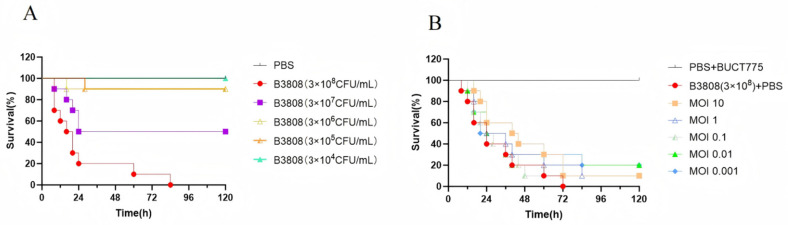
*G. mellonella* larvae infection model and phage therapy: (**A**) Survival rate of the *G. mellonella* larvae infected with strain 3808. Inject *G. mellonella* larvae with 5 µL of B3808 at different concentrations or PBS, incubate at 37 °C, and record the survival rate of the larvae every 12 h. (**B**) Survival rate of the *G. mellonella* larvae treated with phage therapy following B3808 infection.

This study evaluated the in vivo antibacterial effects of phage BUCT775 using a *Galleria mellonella* (*G. mellonella*) larvae infection model. The data showed that as the bacterial inoculum concentration increased, the mortality rate of *G. mellonella* larvae also increased. Within 0–24 h post-infection, the survival rate of *G. mellonella* larvae infected with concentrations of 3 × 10^7^ CFU/mL and 3 × 10^8^ CFU/mL rapidly decreased, with most fatalities occurring during this period (Figure 5A). After 24 h of infection, the survival rate of the larvae infected with concentrations ranging from 3 × 10^5^ CFU/mL to 3 × 10^7^ CFU/mL stabilized. The larvae infected with 3 × 10^7^ CFU/mL experienced 50% mortality within 24 h, while those infected with 3 × 10^4^ CFU/mL showed 100% survival over the 0–120 h period. After treatment with the phages at MOI 0.01 and 0.001, the survival rate of the *G. mellonella* larvae after 120 h increased by 20% compared to the untreated control group (Figure 5B). Furthermore, treatment with phage BUCT775 significantly extended the survival time of the larvae at various concentrations. These results suggest that phage BUCT775 has therapeutic efficacy against 3808 infection.

The evaluation of the in vivo antimicrobial effects of phages using animal infection models is of significant value for their clinical applications [30]. After evaluating the in vivo antibacterial efficacy of phage BUCT775, this study found that the therapeutic effects on *G*. *mellonella* larvae varied depending on the multiplicity of infection (MOI) used. At an MOI of 10, phage BUCT775 demonstrated therapeutic efficacy comparable to that of phage Bφ-R2096 in the *G. mellonella* infection model [31].

### 2.7. Efficacy of Phage BUCT775 in Eliminating Environmental Bacteria

**Figure 6:**
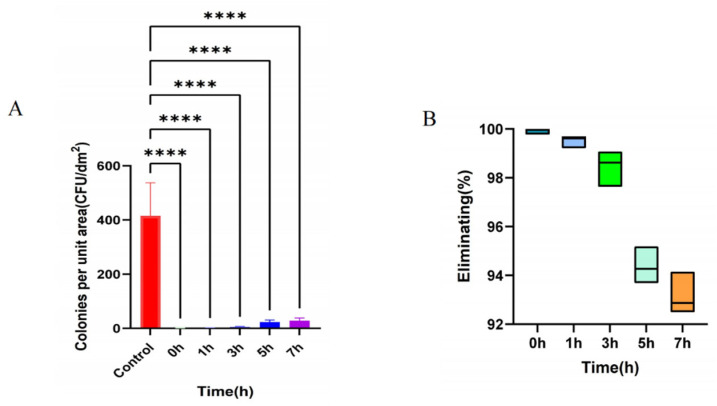
Efficacy of phage BUCT775 in eliminating environmental bacteria: (**A**) The number of *A. baumannii* strain 3808 in the environment following the application of phage spray. The strain count on plates prior to the phage spray application served as the control group. Strain counts were recorded at 0 h, 1 h, 3 h, 5 h, and 7 h after the phage spray application. (**B**) The elimination rate of *A. baumannii* strain 3808 in the environment following the application of the phage spray. Data are presented as mean ± standard deviation. **** *p* < 0.0001.

In hospital environments, inadequate disinfection of surfaces can facilitate the transmission of pathogenic microorganisms, particularly multidrug-resistant (MDR) bacteria, making it crucial to strengthen disinfection protocols to effectively reduce the risk of hospital-acquired infections [32]. The phage BUCT775 spray demonstrated its highest bactericidal efficacy within 0–3 h, achieving an average elimination rate of over 98% (Figure 6A). At 5 h post-application, the bactericidal effect slightly diminished, with the average elimination rate decreasing to 94.38%. However, even at 7 h, the average elimination rate remained above 93% (Figure 6B). These findings indicate that although the antibacterial efficacy of the BUCT775 aerosol may diminish over time, it, nonetheless, retains promising potential as an environmental disinfectant.

Phage-based biocontrol strategies, as an effective means of controlling microbial contamination, have been applied to address numerous challenges in environmental engineering [33]. In previous studies, phage φAB2 demonstrated a >90% elimination efficiency of *A. baumannii* M3237 on hard surfaces within 10 min at a titer of 10^8^ PFU/slide [34]. Phage Abp9, after 2 h of treatment on hard surfaces contaminated with *A. baumannii* Ab9, was able to significantly reduce the colony count by 3–4 orders of magnitude [23]. Compared to these two phages, phage BUCT775 also exhibits the ability to efficiently eliminate host bacteria from the environment in a short period. Potential differences in bacterial removal efficacy on hard surfaces at varying phage titers warrant further investigation in future studies, representing a limitation of the present research.

Phage cocktails, while covering the heterogeneity of bacterial strains, can expand the host range, which is of significant importance for phage therapy and applications [35]. Accelerating the isolation and identification of novel phages will provide more potential options for the design of phage cocktails. Thus, our research provides an effective phage-based strategy for the treatment of *A. baumannii* infections and environmental disinfection.

## 3. Materials and Methods

### 3.1. Bacterial Strains and Culture Conditions

All the *A. baumannii* strains used in this study were isolated from environmental samples from Peking University Third Hospital and Affiliated Hospital of Qingdao University, China. These strains were stored in our laboratory with 50% glycerol in −80 °C, and multipoint sequence typing was identified by whole genome sequencing. All the strains were cultured in Luria–Bertani (LB) broth at 37 °C with shaking (180 rpm) for 6 h to reach the logarithmic stage.

### 3.2. Phage Isolation and Purification

Phage was isolated from a wastewater sample collected from a sewage treatment plant in Beijin using *A. baumannii* strain 3808 as the host. A 20 mL sewage sample was centrifuged at 11,000× *g* for 5 min, followed by filtration through a 0.22 μm filter to remove bacteria and other impurities. The filtrates were mixed with host strain 3808 in LB broth and cultured at 37 °C with shaking (180 rpm) for 12 h. The culture was then centrifuged at 4 °C, 11,000× *g* for 5 min, and the supernatant was filtered using a 0.22 μm filter. A mixture of 100 μL supernatant and 200 μL of the logarithmic-phage host strain was added to LB soft agar and poured onto LB agar plates, and then incubated at 37 °C for 12 h. A single transparent plaque was selected and purified by repeating the soft agar overlay method three times to obtain phages with consistent plaque appearance.

### 3.3. Transmission Electron Microscopy

To visualize the morphology of phage BUCT775, 20 μL of the purified phage at a titer of 1 × 10^9^ PFU/mL was loaded onto a carbon copper grid for 10 min. Then, the sample was stained with 2% phosphotungstic acid (PTA) for 90 s, followed by the removal of excess stain and air-drying at room temperature. The morphology of phage BUCT775 was observed using a JEM-1200EX (JEOL Ltd., Tokyo, Japan) transmission electron microscope at an accelerating voltage of 80 kV. Five well-resolved and complete images were randomly selected to analyze the morphological features and average size of the phage.

### 3.4. Determination of Phage Host Range

To determine the host range of the phage, 15 *A. baumannii* strains from Peking University Third Hospital and the Affiliated Hospital of Qingdao University were used. In total, 200 μL of each strain was added to 5 mL of LB soft agar, mixed thoroughly, and poured onto LB agar. The phage stock was diluted in phosphate-buffered saline (PBS) to concentrations of 10^3^–10^8^ PFU/mL, and 2 μL was spotted onto the bacterial lawn. The plates were incubated at 37 °C for 12 h, and the presence of individual plaques indicated phage susceptibility.

### 3.5. Optimal Multiplicity of Infection (MOI)

The MOI was defined as the ratio of phage particles to host bacteria [36]. A total of 500 μL of host bacteria in the exponential phase were mixed with different dilutions of phages corresponding to MOIs of 10, 1, 0.1, 0.01, and 0.001. After a 15 min incubation at 37 °C, the mixture was added to LB broth and cultured at 37 °C with shaking (180 rpm) for 12 h. The mixture was then centrifuged at 4 °C, 11,000× *g* for 5 min, and filtered using a 0.22 μm filter. Phage titers were determined using the soft agar overlay method. The optimal MOI was determined based on the highest phage titer.

### 3.6. One-Step Growth Curve

In order to accurately evaluate the growth dynamics of the phage BUCT775, a one-step growth curve was performed. In total, 1 mL of phage suspension (1 × 10^6^ PFU/mL) was mixed with 1 mL of bacterial culture (1 × 10^8^ CFU/mL). The mixture was incubated at 37 °C for 10 min for phage adsorption, and then centrifuged at 4 °C, 11,000× *g* for 5 min. The supernatant was discarded, and the bacterial pellets with adsorbed phages were washed three times with PBS. The mixture was resuspended in 50 mL of LB broth and cultured at 37 °C with shaking (180 rpm). A total of 500 μL of culture was collected at various time points (0, 5, 10, 15, 20, 25, 30, 40, 50, 60, 70, 80, 90, 100, 110, 120, 150, and 180 min) and centrifuged, and the phage titer was measured using the soft agar overlay method. Burst size was determined by dividing the increase in the phage titer following lysis by the number of initially infected bacterial cells. Three replicates were performed.

### 3.7. Thermal and pH Sensitivity

The stability of the phage was tested at different temperatures (4, 26, 37, 45, 55, and 65 °C). A total of 100 μL of the phage was incubated at each temperature for 1h and 2 h, and the phage titer was determined by the soft agar overlay method. For pH stability, the phages were incubated at different pH values (2–13) at 37 °C for 1 h, and the phage titers were measured by the soft agar overlay method. Each experiment was conducted in triplicate.

### 3.8. Genomic Sequencing and Bioinformatic Analysis of the Phage

The genomic DNA of the phage was extracted using the λ phage genomic DNA extraction kit (Beijing Regen Biotechnology Co., Ltd., Beijing, China) according to the manufacturer’s instructions. The genomic DNA was sequenced on the Illumina NovaXplus platform by Beijing Novogene Bioinformatics Technology Co., Ltd. (Beijing, China). Data quality was controlled using Trimmomatic v0.36 [37]. After removing low-quality reads, all the reads were assembled using the SPAdes 3.13.0 software [38]. Potential open reading frames (ORFs) were predicted using the online tool RAST (https://rast.nmpdr.org/, accessed on 20 July 2024) [39], and the annotated ORFs were verified using the BLASTp tool from the National Center for Biotechnology Information (NCBI) server against the non-redundant sequence database. A circular genome map was constructed using a custom Python 3.13.2 (Python Software Foundation, https://www.python.org, accessed on 23 July 2024) script and subsequently refined using Adobe Illustrator 2025 (Adobe Inc., San Jose, CA, USA). A phylogenetic analysis was conducted using the online tool ViPTree (https://www.genome.jp/viptree/, accessed on 5 August 2024) [40].

### 3.9. Galleria mellonella Model for In Vivo Phage Therapy

The therapeutic efficacy of this phage was evaluated using the *G. mellonella* larvae model. Larvae weighing 250–300 mg (Tianjin Huiyude Biotechnology Co., Ltd., Tianjin, China) as the animal model [41] were randomly divided into six groups, with 10 larvae in each group. Each larva in the experimental groups was injected with 5 μL of bacterial suspension at concentrations of 3 × 10^4^ CFU/mL, 3 × 10^5^ CFU/mL, 3 × 10^6^ CFU/mL, 3 × 10^7^ CFU/mL, and 3 × 10^8^ CFU/mL. The control group was injected with 5 μL of PBS. After injection, the larvae were cultured at 37 °C in the dark, and their survival was monitored every 4 h. For subsequent therapeutic evaluation, the larvae were inoculated with 5 µL of bacterial suspension (3 × 10^8^ CFU/mL) and, one hour post-infection, treated with 5 µL of phage suspension at varying multiplicities of infection (MOIs of 10, 1, 0.1, 0.01, and 0.001). The larvae were observed every 4 h for signs of mortality, with the larvae showing no movement or turning black recorded as deceased. Three replicates were performed.

### 3.10. Disinfection Assay Using Phage BUCT775

To evaluate the efficacy of phage BUCT775 in eliminating *A. baumannii* in an environmental setting, a sealed plastic chamber (45 × 30 × 25 cm) was used to simulate an indoor environment [42]. Sterilized LB agar plates, with their lids removed, were randomly placed inside the chamber, and 5 mL of bacterial suspension (3 × 10^3^ CFU/mL) was evenly sprayed into the chamber using a sterilized sprayer. After allowing the bacteria to settle for 30 min, one plate was collected to quantify the bacterial counts. Subsequently, the chamber was evenly sprayed with a phage suspension (3 × 10^8^ PFU/mL) using a sterilized sprayer. The plates were collected at 0, 1, 3, 5, and 7 h, and then cultured at 37 °C for 12 h. The experiment was repeated three times.

### 3.11. Statistical Analysis

All data were analyzed using a one-way ANOVA test and Dunnett’s multiple comparisons test in GraphPad Prism 10. The results were presented as the mean and standard deviation (SD). *p* < 0.05 was considered statistically significant. Each experiment was performed in triplicate.

## Figures and Tables

**Table 1:** Host range analysis of phage BUCT775.

Species	Strains	ST Type (Pasteur)	Susceptibility	Efficiency of Plating (EOP)	Origin
*Acinetobacter baumannii*	3808	ST2	+	Host strain	The Affiliated Hospital of Qingdao University
	T1319	ST2	−	0	Peking University Third Hospital
	T2297	ST40	−	0	Peking University Third Hospital
	T2321	ST2	−	0	Peking University Third Hospital
	T2347	ST2	−	0	Peking University Third Hospital
	T2404	ST2	−	0	Peking University Third Hospital
	T2634	ST2	+	0.1	Peking University Third Hospital
	T2673	ST2	−	0	Peking University Third Hospital
	T2747	ST2	−	0	Peking University Third Hospital
	T2784	ST40	−	0	Peking University Third Hospital
	T3153	ST40	−	0	Peking University Third Hospital
	T3311	ST113	−	0	Peking University Third Hospital
	T3640	ST2	−	0	Peking University Third Hospital
	T3666	ST2	+	1	Peking University Third Hospital
	T3789	ST2	+	1	Peking University Third Hospital

+, susceptibility; −, non-susceptibility.

**Table 2:** Features of the key open reading frames (ORFs) of phage BUCT775.

ORF	Strand	Start	Stop	Length (AA)	Predicted Protein Function	Best-Match BLASTp Result	Query Cover	E-Values	Identity	Accession
1	-	436	2	144	tail fiber protein	*Acinetobacter* phage vB_AbaP_B4	100%	4 × 10^−94^	99.31%	WNO29457.1
2	-	3541	443	1032	internal virion protein with endolysin domain	*Acinetobacter* phage Abp1	100%	0.0	99.81%	YP_008058238.1
3	-	6436	3551	961	internal virion lysozyme motif	*Acinetobacter* phage Abp1	100%	0.0	99.79%	YP_008058237.1
4	-	7120	6449	223	internal virion protein	*Acinetobacter* phage Abp1	100%	8 × 10^−159^	100.00%	YP_008058236.1
5	-	9411	7120	763	tail protein	*Acinetobacter* phage SWH-Ab-1	100%	0.0	99.21%	YP_009949054.1
6	-	9980	9420	186	tail protein	*Acinetobacter* phage Abp1	100%	5 × 10^−133^	100%	YP_008058234.1
8	-	10,611	10,426	61	tail protein	*Acinetobacter* phage Abp1	100%	1 × 10^−32^	100.00%	YP_008058232.1
9	-	11,698	10,667	343	capsid protein	*Acinetobacter* phage APK127v	100.00%	0.0	99.42%	URQ05181.1
10	-	12,574	11,714	286	head scaffolding protein	*Acinetobacter* phage Abp1	100.00%	0.0	100.00%	YP_008058230.1
11	-	14,139	12,583	518	head–tail adaptor	*Acinetobacter* phage Abp1	100%	0.0	100%	YP_008058229.1
14	-	17,116	14,699	805	RNA polymerase	*Acinetobacter* phage Abp1	100%	0.0	99.88%	YP_008058226.1
17	-	19,154	18,714	146	endonuclease VII	*Acinetobacter* phage Abp1	100%	5 × 10^−105^	100%	YP_008058223.1
18	-	19,588	19,151	145	HNH endonuclease	*Acinetobacter* phage Abp1	100%	3 × 10^−104^	100%	YP_008058222.1
19	-	20,525	19,569	318	DNA exonuclease	*Acinetobacter* phage SWH-Ab-3	100%	0.0	100.00%	YP_009949090.1
22	-	22,121	21,642	159	HNH endonuclease	*Acinetobacter* phage SWH-Ab-1	100%	8 × 10^−115^	98.74%	YP_009949038.1
23	-	24,430	22,130	766	DNA polymerase	*Acinetobacter* phage SWH-Ab-1	100%	0.0	99.87%	YP_009949037.1
24	-	24,873	24,427	148	HNH endonuclease	*Acinetobacter* phage phiAB1	100%	4 × 10^−104^	100%	YP_009189356.1
25	-	26,111	25,131	326	ATP-dependent DNA ligase	*Acinetobacter* phage SWH-Ab-1	100%	0.0	99.39%	YP_009949035.1
26	-	27,412	26,114	432	DNA helicase	*Acinetobacter* phage SWH-Ab-1	100%	0.0	100%	YP_009949034.1
29	-	28,733	27,978	251	DNA primase	Acinetobacter phage Abgy2021-6-2	100%	0.0	100%	WPF70317.1
30	-	29,212	28,763	149	HNH endonuclease	*Acinetobacter* phage SWH-Ab-3	100%	5 × 10^−107^	100%	YP_009949077.1
46	-	35,819	35,685	44	DNA binding protein	*Acinetobacter* phage IME-200	100%	2 × 10^−21^	100.00%	YP_009216494.1
47	-	37,753	35,816	645	terminase large subunit	*Acinetobacter* phage Abp1	100%	0.0	100.00%	YP_008058244.1
48	-	38,071	37,763	102	terminase small subunit	*Acinetobacter* phage Abp1	100%	2 × 10^−66^	100.00%	YP_008058243.1
49	-	38,688	38,131	185	EF hand domain protein	*Acinetobacter* phage Abp1	100%	8 × 10^−131^	100.00%	YP_008058242.1
50	-	39,010	38,675	111	holin/anti-holin	*Acinetobacter* phage Abp1	100%	3 × 10^−73^	100.00%	YP_008058241.1
52	-	41,003	39,342	553	tail fiber protein	*Acinetobacter* phage SWH-Ab-3	100%	0.0	99.83%	YP_009949108.1

## Data Availability

The genome sequence of BUCT 775 has been submitted to GenBank with accession number PV277657.

## Supplementary Materials

The following supporting information can be downloaded at: https://www.mdpi.com/article/10.3390/ijms26157279/s1.
