# Supplementary material for: Characterization and Complete Genomic Analysis of a Novel Bacteriophage BUCT775 for Acinetobacter baumannii and Its Elimination Efficiency in the Environment
Source: Int J Mol Sci. 2025 Jul 28;26(15):7279. doi: 10.3390/ijms26157279 (PMC12346957; doi:10.3390/ijms26157279)
Supplement: Supplementary file 1 [file ijms-26-07279-s001.zip › ijms-3601147-supplementary.pdf]

Table S1 Features of the open reading frames (ORFs) of phage BUCT775.

ORF	strand	start	stop	Length (AA)	Predicted Protein Function	Best-match BLASTp Result	Query cover	E-values	Identity	Accession
1	-	436	2	144	tail fiber protein	*Acinetobacter* phage vB_AbaP_B4	100%	4×10^{-94}	99.31%	WNO29457.1
2	-	3541	443	1032	internal virion protein with endolysin domain	*Acinetobacter* phage Abp1	100%	0.0	99.81%	YP_008058238.1
3	-	6436	3551	961	internal virion lysozyme motif	*Acinetobacter* phage Abp1	100%	0.0	99.79%	YP_008058237.1
4	-	7120	6449	223	internal virion protein	*Acinetobacter* phage Abp1	100%	8×10^{-159}	100.00%	YP_008058236.1
5	-	9411	7120	763	tail protein	*Acinetobacter* phage SWH-Ab-1	100%	0.0	99.21%	YP_009949054.1
6	-	9980	9420	186	tail protein	*Acinetobacter* phage Abp1	100%	5×10^{-133}	100%	YP_008058234.1
7	-	10,414	10,121	97	hypothetical protein M172_gp41	*Acinetobacter* phage Abp1	100%	1×10^{-55}	100%	YP_008058233.1
8	-	10,611	10,426	61	tail protein	*Acinetobacter* phage Abp1	100%	1×10^{-32}	100.00%	YP_008058232.1
9	-	11,698	10,667	343	capsid protein	*Acinetobacter* phage APK127v	100.00%	0.0	99.42%	URQ05181.1
10	-	12,574	11,714	286	head scaffolding protein	*Acinetobacter* phage Abp1	100.00%	0.0	100.00%	YP_008058230.1
11	-	14,139	12,583	518	head-tail adaptor	*Acinetobacter* phage Abp1	100%	0.0	100%	YP_008058229.1
12	-	14,399	14,148	83	hypothetical protein M172_gp36	*Acinetobacter* phage Abp1	100%	5×10^{-53}	100%	YP_008058228.1
13	-	14,593	14,396	65	hypothetical protein M172_gp35	*Acinetobacter* phage Abp1	100%	1×10^{-39}	100%	YP_008058227.1
14	-	17,116	14,699	805	RNA polymerase	*Acinetobacter* phage Abp1	100%	0.0	99.88%	YP_008058226.1
15	-	17,775	17,125	216	hypothetical protein M172_gp33	*Acinetobacter* phage Abp1	100%	1×10^{-155}	100.00%	YP_008058225.1
16	-	18,710	17,775	311	hypothetical protein	*Acinetobacter* phage vB_AbaP_PD-AB9	100%	0.0	100.00%	YP_009189846.1

ORF	strand	start	stop	Length (AA)	Predicted Protein Function	Best-match BLASTp Result	Query cover	E-values	Identity	Accession
					AU093_gp23					
17	-	19,154	18,714	146	endonuclease VII	*Acinetobacter* phage Abp1	100%	5×10^{-105}	100%	YP_008058223.1
18	-	19,588	19,151	145	HNH endonuclease	*Acinetobacter* phage Abp1	100%	3×10^{-104}	100%	YP_008058222.1
19	-	20,525	19,569	318	DNA exonuclease	*Acinetobacter* phage SWH-Ab-3	100%	0.0	100.00%	YP_009949090.1
20	-	20,677	20,558	39	hypothetical protein M172_gp28	*Acinetobacter* phage Abp1	100%	6×10^{-20}	100%	YP_008058220.1
21	-	21,624	20,734	296	hypothetical protein AU093_gp27	Acinetobacter phage vB_AbaP_PD-AB9	100%	0.0	99.66%	YP_009189850.1
22	-	22,121	21,642	159	HNH endonuclease	*Acinetobacter* phage SWH-Ab-1	100%	8×10^{-115}	98.74%	YP_009949038.1
23	-	24,430	22,130	766	DNA polymerase	*Acinetobacter* phage SWH-Ab-1	100%	0.0	99.87%	YP_009949037.1
24	-	24,873	24,427	148	HNH endonuclease	*Acinetobacter* phage phiAB1	100%	4×10^{-104}	100%	YP_009189356.1
25	-	26,111	25,131	326	ATP-dependent DNA ligase	*Acinetobacter* phage SWH-Ab-1	100%	0.0	99.39%	YP_009949035.1
26	-	27,412	26,114	432	DNA helicase	*Acinetobacter* phage SWH-Ab-1	100%	0.0	100%	YP_009949034.1
27	-	27,661	27,425	78	hypothetical protein M172_gp19	*Acinetobacter* phage Abp1	100%	2×10^{-50}	100.00%	YP_008058211.1
28	-	27,978	27,661	105	hypothetical protein	*Acinetobacter* phage Abgy2021-6-2	100%	1×10^{-69}	100%	WPF70316.1
29	-	28,733	27,978	251	DNA primase	Acinetobacter phage Abgy2021-6-2	100%	0.0	100%	WPF70317.1
30	-	29,212	28,763	149	HNH endonuclease	*Acinetobacter* phage SWH-Ab-3	100%	5×10^{-107}	100%	YP_009949077.1
31	-	29,443	29,234	69	hypothetical protein GGJFMLOI_00027	*Acinetobacter* phage Ab124	100%	9×10^{-42}	97.10%	QMP19146.1

ORF	strand	start	stop	Length (AA)	Predicted Protein Function	Best-match BLASTp Result	Query cover	E-values	Identity	Accession
32	-	29,651	29,433	72	hypothetical protein M172_gp14	*Acinetobacter* phage Abp1	100%	1×10^{-43}	100.00%	YP_008058206.1
33	-	29,839	29,648	63	hypothetical protein M172_gp13	*Acinetobacter* phage Abp1	100%	2×10^{-38}	100.00%	YP_008058205.1
34	-	29,993	29,826	55	hypothetical protein AU093_gp39	*Acinetobacter* phage vB_AbaP_PD-AB9	100%	2×10^{-29}	100.00%%	YP_009189862.1
35	-	30,438	30,004	144	hypothetical protein AU093_gp40	*Acinetobacter* phage vB_AbaP_PD-AB9	100%	2×10^{-102}	100.00%	YP_009189863.1
36	-	30,928	30,440	162	hypothetical protein M172_gp10	*Acinetobacter* phage Abp1	100%	9×10^{-116}	100.00%	YP_008058202.1
37	-	31,405	31,007	132	hypothetical protein PJNENDPG_00018	*Acinetobacter* phage AB_SZL2	99%	1×10^{-91}	99.24%	XCZ58317.1
38	-	31,668	31,564	34	hypothetical protein PJNENDPG_00017	*Acinetobacter* phage AB_SZL2	100%	1×10^{-14}	97.06%	XCZ58316.1
39	-	32,261	31,665	198	hypothetical protein M172_gp06	*Acinetobacter* phage Abp1	100%	7×10^{-146}	99.49%	YP_008058198.1
40	-	32,569	32,336	77	hypothetical protein	*Acinetobacter* phage AbpL	100%	9×10^{-49}	100.00%	UVD42095.1
41	-	33,039	32,665	124	hypothetical protein M172_gp04	Acinetobacter phage Abp1	100%	2×10^{-85}	99.19%	YP_008058196.1
42	-	33,550	33,041	169	hypothetical protein APK128_gp04	*Acinetobacter* phage vB_AbaP_APK128	100%	5×10^{-120}	100.00%	QVD48847.1
43	-	33,904	33,623	93	hypothetical protein M172_gp02	*Acinetobacter* phage Abp1	100%	2×10^{-59}	98.92%	YP_008058194.1
44	-	34,540	34,334	68	hypothetical protein M172_gp01	*Acinetobacter* phage Abp1	100%	9×10^{-43}	100.00%	YP_008058193.1
45	-	35,725	35,522	67	hypothetical protein	*Clostridium butyricum*	100%	7×10^{-36}	100.00%	WP_367736007.1
46	-	35,819	35,685	44	DNA binding protein	*Acinetobacter* phage IME-200	100%	2×10^{-21}	100.00%	YP_009216494.1

ORF	strand	start	stop	Length (AA)	Predicted Protein Function	Best-match BLASTp Result	Query cover	E-values	Identity	Accession
47	-	37,753	35,816	645	terminase large subunit	*Acinetobacter* phage Abp1	100%	0.0	100.00%	YP_008058244.1
48	-	38,071	37,763	102	terminase small subunit	*Acinetobacter* phage Abp1	100%	2×10^{-66}	100.00%	YP_008058243.1
49	-	38,688	38,131	185	EF hand domain protein	*Acinetobacter* phage Abp1	100%	8×10^{-131}	100.00%	YP_008058242.1
50	-	39,010	38,675	111	holin/anti-holin	*Acinetobacter* phage Abp1	100%	3×10^{-73}	100.00%	YP_008058241.1
51	-	39,288	39,058	76	hypothetical protein M172_gp48	*Acinetobacter* phage Abp1	72%	5×10^{-31}	100.00%	YP_008058240.1
52	-	41,003	39,342	553	tail fiber protein	*Acinetobacter* phage SWH-Ab-3	100%	0.0	99.83%	YP_009949108.1
